# Regulation of intestinal epithelial homeostasis by mesenchymal cells

**DOI:** 10.1186/s41232-024-00355-0

**Published:** 2024-09-26

**Authors:** Hisako Kayama, Kiyoshi Takeda

**Affiliations:** 1 Laboratory of Immune Regulation, Department of Microbiology and Immunology, Graduate School of Medicine, Osaka University, Suita, Osaka Japan; 2 WPI Immunology Frontier Research Center, Osaka University, Suita, Osaka Japan; 3 Institute for Advanced Co-Creation Studies, Osaka University, Suita, Osaka 565-0871 Japan; 4 Integrated Frontier Research for Medical Science Division, Institute for Open and Transdisciplinary Research Initiatives, Osaka University, Suita, Osaka Japan; 5 Center for Infectious Disease Education and Research, Osaka University, Suita, Osaka Japan

**Keywords:** Colorectal cancer, Crohn’s disease, Epithelial cell, Inflammatory bowel disease, Intestinal stem cell, Mesenchymal stromal cell, Ulcerative colitis

## Abstract

The gastrointestinal tract harbors diverse microorganisms in the lumen. Epithelial cells segregate the luminal microorganisms from immune cells in the lamina propria by constructing chemical and physical barriers through the production of various factors to prevent excessive immune responses against microbes. Therefore, perturbations of epithelial integrity are linked to the development of gastrointestinal disorders. Several mesenchymal stromal cell populations, including fibroblasts, myofibroblasts, pericytes, and myocytes, contribute to the establishment and maintenance of epithelial homeostasis in the gut through regulation of the self-renewal, proliferation, and differentiation of intestinal stem cells. Recent studies have revealed alterations in the composition of intestinal mesenchymal stromal cells in patients with inflammatory bowel disease and colorectal cancer. A better understanding of the interplay between mesenchymal stromal cells and epithelial cells associated with intestinal health and diseases will facilitate identification of novel biomarkers and therapeutic targets for gastrointestinal disorders. This review summarizes the key findings obtained to date on the mechanisms by which functionally distinct mesenchymal stromal cells regulate epithelial integrity in intestinal health and diseases at different developmental stages.

## Background

The gastrointestinal tract harbors a large number of commensal bacteria, referred to as microbiota, in the lumen, while various types of immune cells are present in the lamina propria. Intestinal epithelial cells form a physical barrier and a chemical barrier, which prevents direct interaction between the microbiota and the immune cells. As the basis for this barrier, a monolayer of intestinal epithelium composed of a variety of differentiated cell types is constantly regenerated from intestinal stem cells located at the crypt bases [1, 2]. The self-renewal, differentiation, and proliferation of intestinal stem cells are precisely manipulated by various secretory factors, such as WNTs, bone morphogenetic proteins (BMPs), and R-spondins. This manipulation is necessary to prevent the excessive growth of intestinal stem cells and the disruption of epithelial tissue regeneration [2].

Intestinal epithelial cells are located above the basement membrane, which is an extracellular matrix compartment [3, 4]. Fibroblasts and myofibroblasts, which are mesenchymal stromal cell populations, play roles in constructing this compartment by secreting various proteins, including collagens, glycoproteins, proteoglycans, and extracellular matrix remodeling-related proteolytic enzymes, such as matrix metalloproteinases (MMPs) [5]. Among these proteins, collagens are involved in the regulation of epithelial elasticity and tensile strength. Meanwhile, glycoproteins and proteoglycans bind to signaling molecules, including amphiregulin, WNTs, BMPs, EGF, and FGF, thereby contributing to epithelial cell survival, proliferation, differentiation, migration, and apoptosis.

Recent advances in single-cell RNA-sequencing (scRNA-seq) and multi-omics technologies have revealed the diversity of mesenchymal stromal cells, such as fibroblast, pericytes, myofibroblasts, and myocyte, within individual tissues, along with the specific functions of these subsets in health and diseases [6–11]. Molecular markers for these cells include PDGFRα, PDGFRβ, vimentin, CD90, podoplanin, collagens, and αSMA, while they lack the expression of markers for hematopoietic cells (CD45), endothelial cells (CD31), and epithelial cells (EpCAM). Vimentin^+^ PDGFRα^+^ fibroblasts, a major cell type among cells producing components of the extracellular matrix, are involved in tissue/organ development and wound healing [12]. Studies have shown that tissue-resident fibroblasts contribute to the maintenance of hematopoietic stem cells [13, 14] and non-hematopoietic stem cells [15–18]. Therefore, perturbations of fibroblast physiology lead to the onset and/or progression of tissue fibrosis, cancer, and chronic inflammatory disorders.

Ulcerative colitis and Crohn’s disease, the main clinical forms of inflammatory bowel disease (IBD), are relapsing and chronic inflammatory disorders of the gastrointestinal tracts. Their incidence and prevalence have been increasing globally [19]. Although the etiology of IBD is largely unknown, accumulating evidence has demonstrated that its pathogenesis is associated with the dysfunction of epithelial barrier integrity, dysregulation of innate/adaptive immune responses, microbiota, environmental factors, dietary habits, and genetics [20, 21]. In the intestine of patients with IBD [22–26], as well as those with colorectal cancer [27–30], there is a change in the composition of mesenchymal stromal cell populations, while immune cell and epithelial cell populations are also altered.

In this review, we provide an overview of the roles of intestinal mesenchymal stromal cells in the establishment and maintenance of epithelial homeostasis in intestinal health and diseases at different developmental stages.

### Functionally distinct cell types in adult intestinal epithelium

**Fig. 1:**
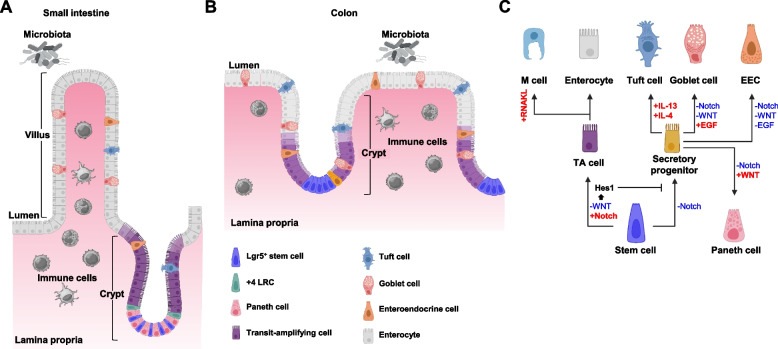
Intestinal epithelial cells and their regulatory signals. **A** In the small intestine, Lgr5^+^ stem cells are intercalated between Paneth cells at the crypt base. These cells continuously give rise to proliferating transit-amplifying (TA) cells, which are present at the remainder of the crypts. The + 4 long-term label-retaining cells (LRCs), which reside at the fourth position from the base of crypts, are quiescent under homeostatic conditions, while they are activated and reconstruct the Lgr5^+^ stem cell compartment during epithelial regeneration after injury. TA cells differentiate into functionally distinct cell types, such as tuft cells, goblet cells, enteroendocrine cells, and absorptive enterocytes. **B** In the colon, Lgr5^+^ stem cells localized at the crypt base generate TA cells, which reside in the lower half part of the crypt and differentiate into multiple cell type, including goblet cells, tuft cells, enteroendocrine cells, and absorptive enterocytes. Homeostatic turnover of epithelial cells occurs every 3–5 days in the small intestine and 5–7 days in the colon. **C** Lgr5^+^ stem cells constantly replenish functionally mature cells under homeostatic conditions. Fate determination and differentiation of Lgr5^+^ stem cell progeny excluded from the stem cell niche are precisely manipulated by various secretory molecules. Dll1^+^ TA (secretory progenitor) cells mature into goblet cells, tuft cells, enteroendocrine cells, and Paneth cells, while Dll1^−^ TA cells supply absorptive enterocytes. Notch signaling promotes enterocyte differentiation and conversely inhibit differentiation of secretory lineage cells by inducing the expression of Hes1. Secretory progenitor cells receiving WNT signal through Eph receptors return to the crypt base and mature into Paneth cells. Meanwhile, secretory progenitor cells differentiate into goblet cells or enteroendocrine lineage cells when they are not supplied with sufficient WNT proteins. Th2 cytokines IL-4 and IL-13 drive tuft cell differentiation. In the epithelium of Peyer’s patches, RANKL derived from mesenchymal cells elicits microfold cell (M cell) differentiation through induction of transcription factor SpiB expression

The epithelial monolayer in the intestine provides the first line of host defense against pathogens. It is composed of diverse cell types [2, 31]: fast-cycling (active) Lgr5^+^ stem cells and slow-cycling (quiescent) + 4 long-term label-retaining cells (LRCs), which have multipotency and self-renewal ability, proliferating transit-amplifying (TA) cells, secretory lineage cells (i.e., Paneth cells whose distribution appears to be limited to the small intestine, goblet cells, tuft cells, and enteroendocrine cells as well as microfold cells present in Peyer’s patches), and absorptive enterocytes, which constitute more than 80% of all differentiated intestinal epithelial cells (Fig. 1A and B). The small intestine is made up of a huge number of crypt-villus units [32], while the luminal surface of colonic crypts is flat and lacks villi [2, 32]. Lgr5^+^ stem cells, + 4 LRCs, and Paneth cells are present at the crypt bases of the intestine, while proliferating TA cells are arranged near these bases. The cells generated from stem cells or their progeny near or at the crypt base migrate toward the villus in the small intestine and the top of the crypt in the colon. In this context, they differentiate and mature into multiple cell types, such as absorptive enterocytes, tuft cells, goblet cells, and enteroendocrine cells.

The intestinal tract harbors multiple microorganisms, including bacteria, viruses, and fungi, as commensals in the lumen [33–37], while adaptive immune cells, IFN-γ-producing T-helper 1 (Th1) cells, IL-17-producing Th17 cells, Foxp3^+^ regulatory T cells, IgA-producing plasma cells, and B cells and innate immune cells, macrophages, eosinophils, neutrophils, dendritic cells, and innate lymphoid cells (ILCs) reside in the lamina propria [38–40]. To segregate the microorganisms from the immune cells, a mucus layer organized by MUC2 secreted by goblet cells [41] serves as a physical barrier in the colon, whereas antimicrobial peptides (e.g., defensins and cathelicidins) and chemical barrier molecules (e.g., Reg3 family proteins) are secreted by enterocytes [42] and Paneth cells [43] in the small intestine. Patients with IBD show exacerbated immune responses in the intestinal mucosa accompanied by perturbations of the epithelial barrier [44–46]. These findings indicate the importance of the precise regulation of epithelial barrier integrity in the intestine to avoid the excessive activation of mucosal immune cells linked to the development of IBD.

### The signaling pathways responsible for the establishment and maintenance of epithelial homeostasis

Approximately, 10^11^ epithelial cells are lost in the human intestine each day [47]. Thus, to maintain homeostasis, Lgr5^+^ intestinal stem cells need to constantly replenish functionally mature cells. A large body of evidence suggests that the balance among quiescence, proliferation, cell fate decision, and differentiation of intestinal stem cells and their progeny is precisely manipulated by various secretory molecules, such as WNTs, the WNT antagonists DKKs, R-spondins (Rspo1-4), Notch ligands, EGFs, BMPs, and the BMP inhibitors Noggin and Gremlins, in order to maintain homeostasis of intestinal epithelium [2, 31, 32, 48] (Fig. 1C).

#### WNT signaling

WNT canonical signaling initiates gene transcription by inducing the formation of a complex of *β*-catenin with TCF/LEF family protein [49, 50] through a frizzled receptor (frizzled1–10) and its co-receptor LRP (LRP5 or LRP6) [51]. This is essential for crypt maintenance by promoting stem cell proliferation as well as maintaining the undifferentiated status of these cells. The WNT signaling pathway induces the expression of Eph receptors in intestinal epithelium [52], including EphB2 and EphB3. Genetic ablation of *EphB2* or *EphB3* has been shown to lead to the mislocalization of Paneth cells and proliferating cells [52, 53], indicating that WNT signaling ensures the proper localization of stem cells, TA cells, and Paneth cells near or at the crypt base by inducing the expression of EphB receptors. R-spondins produced from endothelial cells and mesenchymal stromal cells [15, 54–57] are recruited by the receptor LGR5 and enhance WNT signaling, which ensures intestinal stem cell homeostasis and facilitates epithelial regeneration [56–62]. Lymphatic endothelial cells close to the crypt bottom produce Rspo3, Wnt2, and the extracellular matrix protein reelin [56, 57]. Notably, receptors for reelin, such as VLDLR [57, 63, 64], ApoER2 [57, 64, 65], α3β1-integrin receptor [63, 64, 66], and EphB2 receptor [66, 67], are expressed in myofibroblasts, crypt cells, and enterocytes in mice. It has been shown that the reelin promotes phosphorylation of Disabled-1 in murine intestinal epithelial cells via its receptor [57, 64]. In addition, lack of reelin signaling leads to an increase in the number of proliferating cells within crypt niches [57] and reduction of matured goblet cells [65] in the murine intestine, indicating that reelin plays essential roles in the self-renewal and differentiation of intestinal stem cells.

#### EGF signaling

EGF produced by Paneth cells and mesenchymal cells stimulates stem cell proliferation through its receptors [68]. Among the four EGF receptors (ErbB1–4), the expression of ErbB1 is restricted to stem cells and TA cells within the crypt of the murine small intestine [69]. To successfully culture intestinal organoids, EGF receptor ligand supplementation is required [70], suggesting that non-epithelial cells elicit sufficient activation of the EGF signaling pathway in intestinal stem cells. In addition to ErbB family members, intestinal stem cells express the negative regulator of ErbB signaling Lrig1 [71]. It has been reported that *Lrig1* deficiency in mice facilitates crypt proliferation and increases the numbers of intestinal stem cells and Paneth cells in the small intestine, which are restored to near-normal levels by treatment with ErbB inhibitor [71]. These findings suggest that the EGF-ErbB signaling pathway is tightly regulated to maintain stem cell homeostasis in the intestine.

#### Notch signaling

The Notch ligands Dll1 and Dll4 are secreted from Paneth cells [31]. Notch1 and Notch2 signaling pathways contribute to the first fate decision of intestinal stem cells. These pathways inhibit differentiation into secretory cells by inducing the expression of Hes1, which suppresses transcription of a master regulator gene of secretory lineage cells, *Atoh1* (also known as *Math1*) [72–75], and directs the differentiation of TA cells into absorptive progenitors and ultimately the development of enterocytes.

#### BMP signaling

BMPs are mainly produced by PDGFRα^high^ cells [15, 76, 77] or Col6a1^+^ CD201^+^ CD34^−^ cells [78] in the crypt top of the colon and the villus of the small intestine. These proteins restrict intestinal stem cell proliferation as well as self-renewal. It has been reported that a complex of Smad1 with Smad4, the formation of which is induced by BMP signaling, constrains the stemness of intestinal stem cells by suppressing the expression of stem cell signature genes through recruiting the histone deacetylase HDAC1 [79]. This in turn leads to the formation of villus tip and crypt top in the small and large intestines in mice, respectively. A BMP gradient along the crypt-villus axis restrains cell proliferation and permits broad zonation of goblet cells, enteroendocrine cells, and enterocytes by switching their gene expression patterns [78, 80–84]. Meanwhile, to maintain stem cell properties, the BMP antagonists Gremlins, Noggin, and Chordins are produced by PDGFRα^low^ cells surrounding the crypt bottom [15, 17, 62, 76, 78, 85, 86].

Intestinal stem cells and their progeny pick up signals related to regulation of the WNT and BMP pathways provided by secretory cells in the epithelium [2, 31], endothelial cells [62], glial cells [87], and PDGFRα^+^ fibroblast subsets, including PDGFRα^high^ Foxl1^+^ telocytes [16, 88], PDGFRα^low^ GREM1^+^ trophocytes [15], CD81^−^ PDGFRα^low^ peri-cryptal cells [16], LTβR^+^ PDGFRα^high^ cells [89], GLI1^+^ cells [17], and pericyte-like cells [90]. Among secretory cells, Paneth cells produce Wnt3, Dll1, Dll4, EGF, and Noggin to maintain epithelial stemness in the small intestine, while enteroendocrine cells secrete Wnt3, Dll1, and Dll4 [31]. However, previous studies showed that a lack of secretory lineage cells caused by *Atoh1* deficiency [91, 92] as well as epithelial cell-specific deletion of *Wnt3a* [93] does not affect intestinal stem cell physiology in vivo but does in vitro. Additionally, studies have shown that the main source of secretory factors related to WNT and BMP signaling is non-epithelial cells in the colon [31, 94]. These findings demonstrate that mesenchymal stromal cells, such as fibroblasts, are essential for the maintenance of epithelial stem cells and their differentiation into several functionally distinct cell types in the intestine.

### The interaction between mesenchymal stromal cells and epithelial cells during development of the intestine

**Fig. 2:**
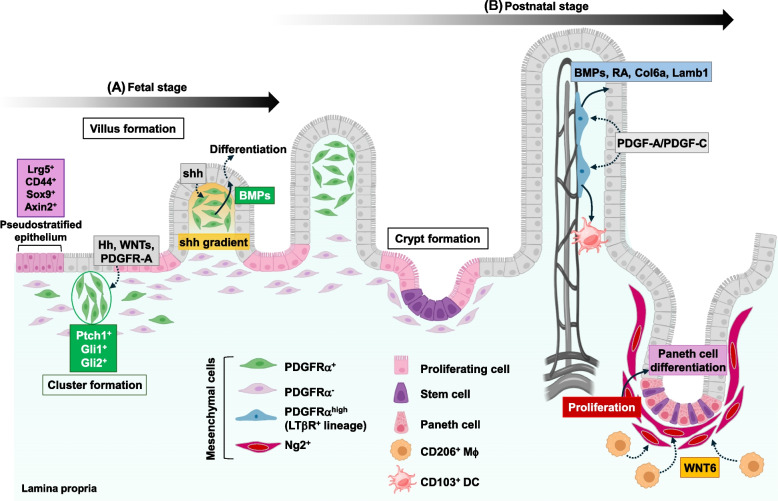
The interplay between epithelial cells and mesenchymal cells in the developing intestine. **A** In the murine fetal intestine, pseudostratified epithelium that expresses stem cell markers (e.g., Lgr5, CD44, Sox9, and Axin2) and mesenchymal cells beneath the epithelium proliferate vigorously from E9.5 to E14.5. The epithelium produces PDGF-A, WNT proteins, and hedgehog (Hh) proteins and elicits the migration and clustering of mesenchymal stromal cells expressing *Pdgfra*, the Hh receptor *Ptch1*, and the transcription factors *Gli1* and *Gli2*. The clustering of mesenchymal cells results in inhibition of cell proliferation in the above epithelial cells. Hh signaling from the epithelium contributes to the continued clustering of mesenchymal cells through induction of the Gli2-dependent transcription of genes related to PCP pathway, which supports vilification. Around E16.5, mesenchymal stromal cells surrounding the villus tip where the shh proteins secreted from epithelial cells are concentrated drive epithelial cell differentiation by producing BMPs, which confines intestinal stem cells to the base of forming villi. Crypt formation in the murine intestine occurs after birth. **B** Maturation of LTβR^+^ PDGFRα^high^ mesenchymal stromal cells that produce BMPs, retinoic acids (RA), collagens, and laminins is induced by epithelial cell-derived PDGF-A and PDGF-C signals at the early stage after birth in the murine intestine, which is essential for reinforcement of the epithelial barrier and regulation of intestinal immune responses in a steady state as well as during tissue regeneration after injury. LTβR^+^ PDGFRα^high^ mesenchymal stromal cell-mediated accumulation of CD103^+^ dendritic cells (DCs) is required for induction of Foxp3^+^ regulatory T-cell development in the lamina propria. In the murine postnatal intestine, CD206^+^ macrophages, which are differentiated from monocytes in a microbiota-dependent fashion, facilitate the proliferation of pericyte marker Ng2^+^ mesenchymal cells through Wnt6 production. Ng2^+^ mesenchymal cells secrete BMP inhibitor Gremlin 1 and induce functionally mature Paneth cells, which is required for prevention of neonatal necrotizing enterocolitis-like pathology in the murine intestine

Epithelial cells communicate bidirectionally with mesenchymal stromal cells in the developing intestine (Fig. 2). Endodermal pseudostratified epithelium and splanchnic mesoderm-derived mesenchymal cells beneath the epithelium proliferate vigorously from embryonic day 9.5 (E9.5) to E14.5 in mice (corresponding to weeks 4 to 9 of gestation in humans), which enables elongation of the gut tube at this stage [95]. The entire pseudostratified epithelium in the murine fetal intestine proliferates and expresses stem cell markers, such as Lgr5, CD44, Sox9, and Axin2 [96, 97], although proliferative cells are present with an apparently random distribution within the pseudostratified epithelium in the human fetal intestine [98]. A murine study showed the expansion of fibroblast/myofibroblast progenitors with the expression of *Mki67*, *Dek*, *Cdk1*, *Aurkb*, and *Hmmr* in the intestine at E14.5 [99], which might be identical to proliferative mesenchymal cells residing in the human fetal intestine [100]. During the same period, the pseudostratified epithelium in the small intestine begins to form villi and construct columnar epithelium [94, 101, 102]. Villus morphogenesis is strongly dependent on the interplay between PDGFRα^+^ cells and the epithelium [94, 95, 101]. The epithelium produces the PDGF ligand PDGF-A and hedgehog (Hh) proteins, such as sonic hedgehog (Shh) and Indian hedgehog, and elicits the migration and clustering of mesenchymal cells that highly express *Pdgfra*, the Shh receptor *Ptch1* (*Ptc1*), and the transcription factor *Gli1*, underneath the sites where villi subsequently emerge [97, 103–106]. It has been reported that a lack of Hh signaling [106] or PDGF/PDGFR signaling [103] perturbs villus formation and also impairs the proliferation of mesenchymal progenitors at the embryonic stage in mice. The activation of Hh signaling in PDGFRα^+^ cells directs the Gli2-dependent transcription of genes related to the planar cell polarity (PCP) pathway [107], such as cadherins *Fat4* and *Dchs1*, *Wnt5a*, and PCP signaling core factors *Vangl1* and *Vangl2* [108]. Both Fat4-Dchs1 signaling and Wnt5a-Vangl2 signaling are essential for the cluster formation of mesenchymal stromal cells and proper vilification in the murine intestine. Therefore, perturbations of these signaling pathways cause abnormal morphogenesis of villi in mice at E15.5, such as shortened and fused villi. The transcription factor Foxl1, which is specifically expressed in intestinal PDGFRα^+^ cells, is encoded by one of the Hh-Gli2 axis-dependent genes [109]. Defect of the *Foxl1* gene leads to delayed villus formation and a decreased number of villi in mice at E14.5 to E16.5 [110], suggesting that the activation of Foxl1-dependent transcription in PDGFRα^+^ cells via the Hh signaling pathway is crucial for epithelial remodeling to form villi in the small intestine.

In the late embryonic developmental stage (around E16.5) in mice, the clustering Ptc1^+^ PDGFRα^+^ cells surrounding the villus tip where epithelium-derived Shh proteins are concentrated drive epithelial cell differentiation by producing BMPs through activation of the Hh signaling pathway [97, 105], which confines intestinal stem cells to the base of forming villi. In human fetal intestine, proliferating cells are restricted to the crypt base and intervillus domain by gestational week 12 [98]. Morphogenesis and maturation of human intestinal epithelium are completed by birth, whereas these processes continue into the neonatal stage in mice, and the mature intestinal epithelium is established by postnatal day 28 [111]. In human fetal small intestine from weeks 7 to 21 of gestation, mesenchymal cells residing in the muscularis mucosa, which is close to the base of crypts, produce WNTs and R-spondins to maintain intestinal stem cells, while PDGFRα^high^ F3^high^ DLL1^high^ subepithelial mesenchymal cells along the crypt-villus axis promote secretory lineage cell differentiation by producing the EGF family member Neuregulin1 [112]. Similarly, Neuregulin1 is secreted from PDGFRα^high^ cells in the villus and crypt top of the murine small intestine [113] and large intestine [76], which is elevated during epithelial regeneration following irradiation-mediated injury [113]. Furthermore, perivascular PDGFRα^high^ cells generated from postnatal LTβR^+^ cells after birth in mice [89] induce maturation of the epithelial barrier and immune system by producing BMPs, retinoic acids, collagens, and laminins in response to epithelial PDGF-A and PDGF-C [89, 114].

Neonatal necrotizing enterocolitis (NEC) is a serious gastrointestinal disorder with a high mortality rate in premature infants [115, 116]. Several lines of evidence have shown that antibiotic use and consequent dysbiosis, such as expansion of Enterobacteriaceae, are associated with the incidence and pathogenesis of NEC [117–121]. A study demonstrated that antibiotic treatment-induced dysbiosis in murine neonates exacerbates NEC-like pathogenesis accompanied by a reduction in Paneth cells [90]. This study also showed that the colonization of *Lactobacillus rhamnosus* induces Paneth cell differentiation and partially improves NEC-like intestinal pathology. Heat-killed *Lactobacillus* promoted the differentiation of monocytes into CD206^+^ macrophages, and Wnt6 derived from CD206^+^ macrophages promoted the proliferation of mesenchymal stromal cells that express mRNA of pericyte marker *Ng2* with *Pdgfra*, *Foxl1*, *Grem1*, and *Cd81 *in vitro. In intestinal epithelial organoid culture, CD206^+^ macrophages and Ng2^+^ mesenchymal stromal cells induced the maturation of Paneth cells. This suggests that interaction among microbiota, immune cells, mesenchymal stromal cells, and epithelial cells during early postnatal development is important for ensuring epithelial barrier integrity, leading to the prevention of intestinal diseases. Taken together, these findings indicate that the bidirectional association between epithelial cells and mesenchymal stromal cells is needed for proper intestinal development and the consequent maintenance of gut homeostasis.

### *Heterogeneity of PDGFRα*^*high*^* fibroblasts and their functions in the maintenance and differentiation of intestinal stem cells*

**Fig. 3:**
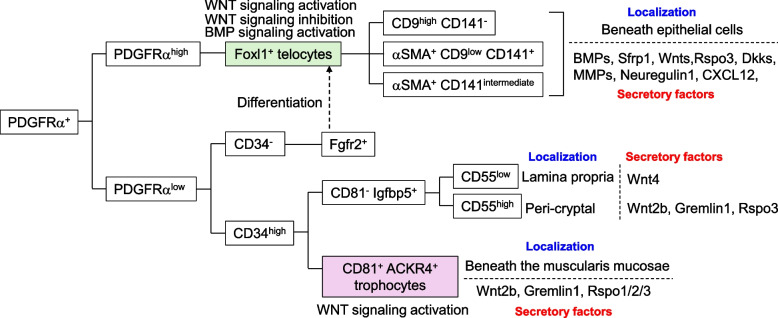
The subsets of PDGFRα^+^ fibroblast in the intestine. PDGFRα^+^ fibroblasts comprise four subsets, including PDGFRα^high^ Foxl1^+^ telocytes, PDGFRα^low^ CD34^high^ CD81^+^ACKR4^+^ trophocytes, PDGFRα^low^ CD34^high^ CD81^−^ Igfbp5^+^ (small intestine) or CD90^+^ (large intestine), and PDGFRα^low^ CD34^−^ Fgfr2^+^ cells, in the intestine of adult mice. Trophocytes lying beneath the muscularis mucosae activate WNT signaling in intestinal stem cells through producing WNT signaling agonist R-spondin 1–3, the canonical WNT ligand Wnt2b, and the BMP inhibitor Gremlin 1, thereby maintaining stemness of intestinal stem cells. CD81^−^ CD55^low^ cells secrete noncanonical WNT ligand Wnt4 in the lamina propria near or at the crypt top. Meanwhile, peri-cryptal CD81^low^ CD55^high^ cells produce intestinal stem cell niche factors, such as R-spondins, WNTs, Gremlins, and Noggin. Telocytes, which are predicted to originate from PDGFRα^low^ CD34^−^ Fgfr2^+^ cells, contain three subsets: CD9^high^ CD141^−^ cells with expression of Neuregulin1, αSMA^+^ CD9^low^ CD141^+^ cells with high expression of CXCL12, and αSMA^+^ CD141^intermediate^ cells expressing Wnt4. These cells are present beneath epithelial cells and contribute to the self-renewal and proliferation of intestinal stem cells as well as differentiation of epithelial cells through modulation of the WNT and BMP signaling pathways by producing various molecules, including WNTs, Rspo3, the canonical WNT signaling antagonists Dkks, WNT inhibitor Sfrp1, BMPs, and MMPs

**Fig. 4:**
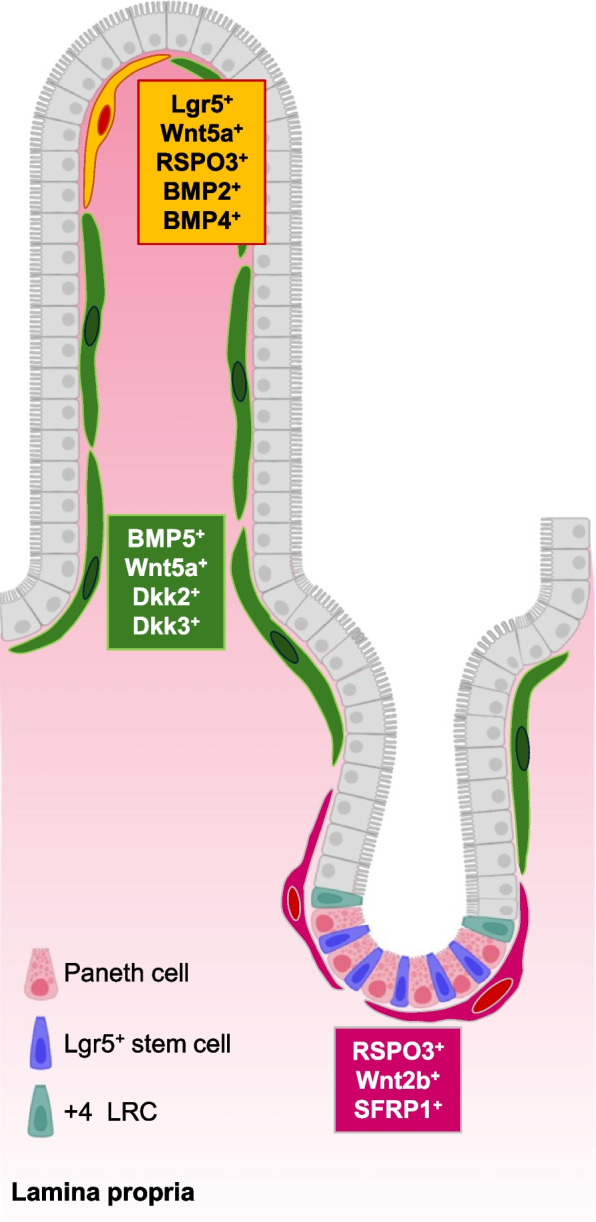
Heterogeneity of PDGFRα^high^ Foxl1^+^ telocytes in the intestine. PDGFRα^high^ Foxl1^+^ telocytes are located beneath epithelial cells along the crypt-villus axis in the small intestine. These cells compartmentalize production of signaling molecules. At the crypt bases, telocytes function as one of key components of intestinal stem cell niche through secretion of WNT signaling amplifier Rspo3 and canonical WNT ligand Wnt2b, and they also produce WNT antagonist Sfrp1. Meanwhile, telocytes distributed in the upper portion of the crypt as well as villus produce the noncanonical WNT ligand Wnt5a, BMP5, and the canonical WNT signaling antagonists Dkk2 and Dkk3. In the villus tip, Lgr5^+^ telocytes supply Wnt5a, Rspo3, BMP2, and BMP4 and contribute to terminal differentiation of enterocytes that express *Ada*, *Nt5e*, *Slc28a2*, *Klf4*, *Cdh1*, and *Egfr*

A recent study [18] showed that large and small intestinal PDGFRα^+^ fibroblasts are divided into four subsets, including PDGFRα^high^ Foxl1^+^ cells, PDGFRα^low^ CD81^+^ CD34^high^ ACKR4^+^ trophocytes, PDGFRα^low^ Fgfr2^+^ CD34^−^ cells, and CD81^−^ CD34^high^ Igfbp5^+^ (small intestine)/CD90^+^ (large intestine), in adult mice (Fig. 3). It also predicted that PDGFRα^high^ Foxl1^+^ cells originate from PDGFRα^low^ Fgfr2^+^ CD34^−^ cells. Additionally, this study identified three PDGFRα^high^ Foxl1^+^ subsets: Neuregulin1^+^ CD9^high^ CD141^−^ cells, CXCL12^+^ αSMA^+^ CD9^low^ CD141^+^ cells, and Adamdec1^+^ Wnt4a^+^ αSMA^+^ CD141^intermediate^ cells. PDGFRα^high^ Foxl1^+^ cells present in the developed intestine, which are referred to as both telocytes and αSMA-expressing myofibroblasts, are one of the most investigated cell types of the intestinal mesenchymal niche [16, 77, 88, 114, 122–128] (Fig. 4). Telocytes are characterized by thin cytoplasmic extensions and are present beneath epithelial cells along the crypt-villus axis in the small intestine, as well as the colonic crypt axis, in a steady state [77, 88, 124]. A study showed that telocytes residing in the crypt base of the murine intestine express the canonical WNT ligand *Wnt2b*, WNT signaling agonist R-spondin *3*, and WNT inhibitor *Sfrp1*, whereas the cells that express the noncanonical WNT ligands *Wnt5a* and *Bmp5* and the canonical WNT signaling antagonists *Dkk2* and *Dkk3* are enriched toward the villus tip in the small intestine [88]. Another study identified that Lgr5^+^ telocytes in the villus tip secrete Wnt5a, Rspo3, BMP2, and BMP4 [77]. Ablation of Lgr5^+^ telocytes in mice suppresses the expression of enterocyte genes in the region toward the villus tip, including *Ada*, *Nt5e*, *Slc28a2*, *Klf4*, *Cdh1*, and *Egfr* [77]. This suggests that villus tip telocytes are essential for the terminal differentiation of enterocytes.

*Foxl1-cre*; *Wnt5a*^flox/flox^ mice exhibit an increased number of apoptotic epithelial cells in the fetal intestine and a short small intestine at birth [123], which might provide some insight into the mechanism underlying the development of short bowel syndrome [129]. Additionally, *Foxl1-creERT2*; *Porcn*^flox/Y^ mice, in which Wnt secretion is ablated in Foxl1-expressing cells specifically following tamoxifen treatment, show abnormal epithelial architecture in the small and large intestines accompanied by declines in epithelial stem and TA cell proliferation [88]. Similarly, in *Foxl1-hDTR* mice, depletion of Foxl1-expressing cells by *diphtheria* toxin results in the disruption of epithelial tissue, such as shorter villi, reduced numbers of Ki67^+^ proliferating cells and Olfm4^+^ stem cells, and crypt collapse in the small intestine [124]. These findings indicate that telocytes function as the cellular source of Wnt4, Wnt5a, and Wnt2b surrounding the stem cell compartment, which is linked to the maintenance of epithelial homeostasis. In the small intestinal crypts of *Foxl1*^−/−^ mice, the number of Ki67^+^ proliferating cells is increased [110], while the expression of BMP2 and BMP4 is decreased in mesenchymal stromal cells. Additionally, lack of the *Foxl1* gene in *APC*^Min/+^ mice was reported to lead to intestinal tumor progression [126]. A murine study also showed that Foxl1 transcriptionally upregulates the expression of BMP4 in telocytes [130]. These findings suggest that Foxl1-dependent transcription of the genes encoding BMPs in telocytes is essential for the regulation of intestinal epithelial cell proliferation, which might be associated with prevention of the intestinal tumorigenesis.

In the human [22] and murine [18] intestines, mesenchymal cells express the chemokine CXCL12. A recent study showed that the transcription factors FOXC1 and FOXC2 elicit CXCL12 expression in blood vascular endothelial cells adjacent to small intestinal crypts after ischemia–reperfusion injury in mice, which induces Rspo3 expression via CXCR4 in lymphatic endothelial cells and thereby promotes tissue regeneration through activation of the WNT signaling pathway in intestinal stem cells [131]. It has been reported that the CXCL12-CXCR4 pathway is involved in immune cell differentiation [13, 14, 132–136] and migration [137–141]. In addition to immune cells, CXCR4 is expressed in intestinal epithelial cells [142]. We found that telocytes without the expression of FOXC1 and FOXC2 highly express CXCL12 in the large and small intestines, and that *Cxcl12* deficiency in Foxl1-expressing cells results in the progression of intestinal tumorigenesis in *APC*^Min/+^ mice (unpublished). Taking these findings together, the induction of functional telocytes is essential for the prevention of intestinal neoplasia as well as the maintenance of epithelial homeostasis.

### *The function of PDGFRα*^*low*^* populations as a key component of the intestinal stem cell niche*

**Fig. 5:**
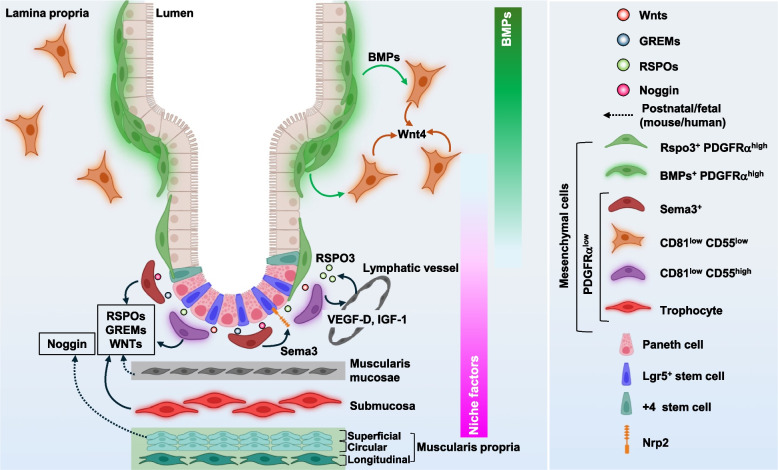
The diversity of PDGFRα^low^ fibroblasts in the intestine. PDFGRα^low^ Gli1^+^ CD90^+^ fibroblasts in the intestine can be divided into CD34^high^ CD81^high^ ACKR4^high^ trophocytes and CD34^high^ CD81^low^ subset. The latter comprises CD55^low^ cells and CD55^high^ cells. PDFGRα^low^ CD55^low^ cells localize in the lamina propria near or at the crypt top and produce noncanonical WNT ligand Wnt4. BMP signaling from telocytes facilitates expression of *Wnt4* in PDGFRα^low^ CD81^−^ cells via the BMP receptors Bmpr1 and Bmpr2, while it inhibits the expression of intestinal stem cell niche factors *Rspo3* and *Grem1*. Trophocytes, CD81^low^ CD55^high^ cells, muscularis mucosae cells, and superficial muscle cells in the muscularis propria cells secrete intestinal stem cell niche factors, including R-spondins, WNTs, Gremlins, and Noggin. In addition, Gli1^+^ CD90^+^ peri-cryptal mesenchymal cells produce Sema3 as well as niche factors and accelerate intestinal stem cell proliferation via the receptor Nrp2. Lymphatic endothelial cells reside close to Gremlin 1^+^ Rspo3^+^ mesenchymal cells at the crypt base in the intestines, and they produce Rspo3. Gremlin 1^+^ Rspo3^+^ mesenchymal cells maintain lymphatic endothelial cells by secreting vascular endothelial cell growth factor D (VEGF-D) and IGF-1 under homeostatic conditions, and they induce expansion of lymphatic endothelial cells by producing VEGF-D, IGF-1, FGF2, IL-6, ANGPTL4, and epiregulin after injury, which support the maintenance of epithelial homeostasis

PDFGRα^low^ Gli1^+^ CD90^+^ fibroblasts include CD34^high^ CD81^+^ ACKR4^+^ trophocytes and CD34^high^ CD81^−^ cells [15–18] (Fig. 5). In mice, trophocytes lying beneath the muscularis mucosae secrete Rspo1, Rspo2, Rspo3, Wnt2b, and the BMP inhibitor Gremlin 1, and thus, they are alone able to maintain the growth of intestinal crypt organoids [15, 16, 114]. In addition to trophocytes, muscularis mucosa cells supply Rspo3, Gremlin 1, and Gremlin 2 in human and murine intestine at the postnatal stage [114]. *Diphtheria* toxin-induced ablation of smooth muscle in the murine intestine at postnatal day 14, which leads to the loss of muscularis mucosae and muscularis propria containing Noggin-producing cells residing underneath trophocytes, is associated with impaired crypt fission and a reduced number of intestinal stem cells through the facilitation of BMP signaling [114]. In this period, trophocytes do not support the growth of small intestinal crypt organoids without growth factors, unlike adult cells. These findings suggest that the layered structure composed of muscularis mucosa cells, trophocytes, and muscularis lamina propria cells becomes established as a complex stem cell niche in the developing intestine.

The PDFGRα^low^ CD34^high^ CD81^−^ population comprises CD55^low^ cells and CD55^high^ cells in the colon [16]. PDFGRα^low^ CD55^low^ cells localize in the lamina propria near or at the crypt top and highly express *Wnt4* and the BMP-inducible gene *Id1.* Peri-cryptal PDFGRα^low^ CD55^high^ cells exhibit the upregulated mRNA expression of *Grem1*, *Rspo3*, and *Wnt2b*. BMP signaling derived from telocytes distributed in the upper portion of the colonic crypt suppresses the expression of intestinal stem cell niche factors Rspo3 and Gremlin 1 and facilitates the expression of Wnt4 in PDGFRα^low^ CD81^−^ cells with expression of the BMP receptors Bmpr1 and Bmpr2 [16], suggesting that the heterogeneity of PDGFRα^low^ CD81^−^ cells is determined by their distance from telocytes, but not factors intrinsic to these cells. *Diphtheria* toxin-mediated elimination of Grem1^+^ cells results in the development of edema-like dilation in the small intestine accompanied by reduced numbers of intestinal stem cells and Paneth cells, increased numbers of TA cells and goblet cells, and inflammatory cell infiltration due to the loss of a low-BMP environment at the crypt base [15]. In addition to signaling molecules related to the WNT and BMP pathways, Sema3 secreted from PDFGRα^low^ Gli1^+^ CD90^+^ cells surrounding the crypt base promotes intestinal stem cell proliferation via the receptor Nrp2 [143]. Prostaglandin E_2_ (PGE_2_) secreted from peri-cryptal PDFGRα^low^ Fgfr2^+^ Rspo1^+^ cells induces Sca-1^+^ epithelial stem cells with a tumorigenic program through YAP/TAZ activation via the receptor Ptger4, which is linked to early tumor progression [144].

PDFGRα^low^ cells modulate immune cell physiology. Intestinal myofibroblasts, which are characterized as PDFGRα^low^ αSMA^high^ cells [22], suppress CD4^+^ T-cell proliferation through the TLR4-dependent induction of PD-L1 expression [145, 146]. PDFGRα^low^ CD34^+^ cells transiently increase expression of the adhesion molecule VCAM-1 as well as the inflammatory mediators CCL2 and Csf1 at the acute phase of dextran sodium sulfate-induced colitis in mice, which attracts immune cells to the mucosa [86]. During colitis, the expression of lymphocyte survival/proliferation factor IL-7 and chemokine CCL19 persists in PDFGRα^low^ CD34^+^ cells at the chronic phase, which contributes to induction of the prolonged survival of T cells [86]. Although the impacts of PDGFRα^low^ cell reprogramming on gut homeostasis and disease pathophysiology require further evaluation, the coordination of PDGFRα^low^ CD34^+^ cell populations with distinct functions and distributions as well as PDGFRα^high^ fibroblasts might be required for epithelial homeostasis and regeneration through maintenance of the intestinal stem cell niche.

### The roles of fibroblasts in IBD pathogenesis

Damage to the epithelium drives a complex process of degradation and biosynthesis of the extracellular matrix to regenerate the epithelial layer under physiological conditions [147–149]. In the gut of patients with IBD, epithelial regenerative ability as well as the epithelial barrier is impaired [150–152], which accelerates inflammatory responses through activating the uptake of luminal antigens by dendritic cells and macrophages in the mucosa. The migratory potential of fibroblasts in the colonic mucosa is known to be reduced in patients with Crohn’s disease and ulcerative colitis [153, 154]. However, proliferation and collagen secretion in intestinal fibroblasts are facilitated in IBD patients [155]. A study showed that Grem1^+^ Grem2^+^ myofibroblasts highly express *COL18A1* and *COL23A1* mRNA in the ileum of patients with Crohn’s disease [156]. Myofibroblast reprogramming into Grem1^+^ Grem2^+^ cells is induced by CHMP1A, TBX3, and RNF168 [156], which might contribute not only to wound healing but also fibrosis in Crohn’s disease [157]. Imbalanced MMP proteolytic activity in the extracellular matrix is associated with failure of wound healing through an excess of extracellular matrix deposition. MMP-2 and MMP-9 promote fragmentation of the extracellular matrix and thereby impair reepithelialization [158]. An increased concentration of MMP-9 is observed in the plasma from patients with active ulcerative colitis [159] and active Crohn’s disease [160]. Additionally, elevated concentrations of MMP-2 and MMP-9 in the plasma positively correlate with high intestinal permeability in ulcerative colitis patients [161]. The expression of MMP-7, which increases epithelial barrier permeability through posttranscriptional downregulation of Claudin 7 in vitro, is upregulated in the colon of ulcerative colitis patients [162]. MMP-13, which promotes goblet cell loss, destabilizes tight junctions, and increases intestinal permeability in mice [163], is elevated at the protein level in the intestinal mucosa of patients with ulcerative colitis and Crohn’s disease [164]. Meanwhile, TNF-α stimulation drives the expression of MMP-1, MMP-3, and TIMP1 and consequently initiates the production of type I and IV collagens in myofibroblasts from the murine [165, 166] and human intestines [167]. *TNF*^∆ARE/+^ mice, in which TNF-α is constitutively overproduced, exhibit the spontaneous development of Crohn’s disease-like ileitis [168]. Additionally, *ColVI*-cre; *TnfRI*^floxneo/floxneo^*TNF*^∆ARE/+^ mice, in which TNF receptor 1 is specifically expressed in intestinal mesenchymal stromal cells, spontaneously develop ileal inflammation [169] while *TNF*^∆ARE/+^ mice with constitutive knockout of *TnfRI* do not. Intriguingly, treatment with an acellular extracellular matrix derived from the porcine small intestine was shown to effectively close anorectal fistulas in patients with Crohn’s disease [170, 171] and promote cecal wound healing in rats [172]. Furthermore, ECM hydrogel therapy was found to ameliorate dextran sodium sulfate-induced colitis in rats through the inhibition of TNF-α production by macrophages [173]. These findings indicate that intestinal mesenchymal stromal cells are one of the target cell types for TNF-α in the onset and/or progression of IBD.

Toll-like receptors (TLRs) recognize pathogen-associated molecular patterns, which are conserved structures on the surface of microbes, and damage-associated molecular patterns [174]. In addition to epithelial cells and immune cells, human CD90^+^ fibroblasts [175] and murine fibroblasts [176] in the intestine also express TLRs. Activation of the TLR-adaptor molecule MyD88/TRIF signaling pathway initiates the expression of pro-inflammatory mediators. MAP3K8, known as Tpl2, is activated through the TLR, IL-1R, and TNFR signaling pathways. It has been shown that Tpl2 kinase-mediated activation of the Cox-2/PGE_2_ pathway in intestinal myofibroblasts, which is initiated by macrophage-derived IL-1β as well as TLR4 ligand lipopolysaccharide, is essential for homeostatic responses in the epithelium through promoting epithelial cell proliferation in mice during dextran sodium sulfate-induced colitis [177]. In ileal myofibroblasts from Crohn’s disease patients, the expression level of *MAP3K8* mRNA was shown to be reduced [177]. Additionally, COX2-expressing CD45^−^ CD44^+^ mesenchymal stromal cells were found to migrate to wound sites of the epithelium via a MyD88-dependent mechanism in mice following dextran sodium sulfate administration and accelerate epithelial tissue regeneration [178]. Furthermore, tissue-resident Vimentin^+^ mesenchymal stromal cells secrete PGE_2_ via the TLR4-Tpl2-COX2 pathway in the propagation phase of dextran sodium sulfate-induced colitis in mice and alleviate pathology of the large intestine [179]. These findings demonstrate that intestinal mesenchymal stromal cells are involved in the maintenance of gut homeostasis and tissue regeneration as well as the pathogenesis of IBD.

Excessive accumulation of immune cells is known to occur in the mucosa of patients with IBD [23, 24, 156, 180, 181]. These infiltrating immune cells produce various pro-inflammatory cytokines, including TNF-α, IL-6, IL-1, IL-23p19, IL-12p40, IFN-γ, and IL-17 [23], which initiates tissue damage and chronic inflammation. Therefore, drugs targeting these cytokines and their downstream signaling molecules (e.g., the JAK family members) are clinically approved or being evaluated for IBD therapy [182, 183]. In patients with IBD, the concentration of TNF-α is elevated in the plasma and the intestinal mucosa. TNF-α produced by various types of cells, including immune cells, adipocytes, fibroblasts, and endothelial cells, promote inflammation through chemotaxis [184, 185], proliferation and activation of immune cells [186–189], degradation of the extracellular matrix [166], and angiogenesis [190] in the intestine. The fact that therapies with anti-TNF antibodies (infliximab and adalimumab, approved for ulcerative colitis and Crohn’s disease; certolizumab pegol, approved for Crohn’s disease; and golimumab, approved for ulcerative colitis) are effective for IBD underscores that TNF plays a crucial role in the progression of this disease [191]. A previous study demonstrated a positive correlation between the numbers of apoptotic mucosal cells and the therapeutic efficacy of infliximab in patients with active Crohn’s disease [192]. Accordingly, the anti-TNF agents infliximab, adalimumab, and certolizumab pegol induce the apoptosis of TNFR2-expressing T cells isolated from IBD patients by binding to transmembrane TNF-α (mTNF-α)-expressing CD14^+^ macrophages [193]. These findings suggest that the induction of T-cell apoptosis through inhibition of the TNFR2-mTNF-α pathway is one of the mechanisms by which anti-TNF agents control intestinal pathology in patients with IBD. Although the use of anti-TNF agents improves the quality of life of IBD patients by promoting mucosal healing [194], 10–40% of IBD patients do not respond to primary anti-TNF therapy [195–198]; moreover, a subset of responders lose their responsiveness to anti-TNF treatment [199–201]. One study showed that expression of the cytokine oncostatin M is increased in inflamed sites of the mucosa of patients with Crohn’s disease and ulcerative colitis; it also revealed that a high level of oncostatin M expression prior to treatment is associated with the failure of anti-TNF therapy [202]. CD45^−^ CD31^−^ EpCAM^−^ mesenchymal stromal cells in the mucosa of patients with IBD express the receptor for oncostatin M (OSMR) and produce pro-inflammatory cytokines, such as IL-1α, IL-1β, IL-6, and IL-17A, and chemokines in response to oncostatin M [202]. In patients with ulcerative colitis, anti-TNF resistance signature is enriched in IL-24^+^ IL-13A2^+^ IL-11^+^ inflammation-associated fibroblasts, monocytes, and type2 conventional dendritic cells [23]. Intriguingly, inflammation-associated fibroblasts highly express OSMR, while inflammatory monocytes express oncostatin M. Similarly, activated mononuclear phagocytes that produce oncostatin M, TNF-α, IL-1α, and IL-1β in inflamed sites of the ileal mucosa of Crohn’s disease patients [24] are associated with resistance to anti-TNF therapy. These findings suggest that cross talk between monocytes and fibroblasts via the oncostatin M-OSMR axis is involved in resistance to anti-TNF therapy in IBD patients.

### The roles of fibroblasts in the intestinal tumor environment

In human lung cancer, group 3 ILCs (ILC3s) were found to contribute to antitumor immunity by supporting the formation of the tertiary lymphoid structure (TLS) composed of a network of CD21^+^ follicular dendritic cells surrounded by T cells and CD21^+^ B cells through the expression of TNF-α, LTα, and LTβ [203]. We found that NKp44^+^ ILC3s highly express *LTA*, *LTB*, and *TNF* in the tumors of patients with colorectal cancers at the T1 or T2 stage, as well as normal colon, and that these cells induce the expression of lymphoid structure formation-related chemokines (e.g., CXCL13, CCL9, and CCL21) and adhesion molecules (e.g., VCAM-1 and ICAM-1) in CD90^+^ mesenchymal stromal cells [204]. In T3/T4 colorectal cancer, the number of NKp44^+^ ILC3s and the expression of *LTA*, *LTB*, and *TNF*, but not ILC3-related molecules (e.g., RORC, IL22, and IL17A), in these cells were decreased. Accordingly, the expression of CXCL13, CCL21, ICAM-1, and VCAM-1 was drastically reduced in CD90^+^ cells within T3/T4 tumor tissues. We found that TLSs are more abundant in the early stages of colorectal cancer, and their number gradually declines as the tumor progresses, indicating that the decreasing number of NKp44^+^ ILC3s expressing *LTA*, *LTB*, and *TNF* mRNAs correlates with the density of TLSs during colorectal cancer progression. These findings suggest that NKp44^+^ ILC3-mediated CD90^+^ mesenchymal stromal cell reprogramming contributes to prevent tumor progression through the induction of TLS formation.

Mesenchymal stomal cells producing the WNT signaling amplifier Rspo3 are increased during intestinal epithelial regeneration to maintain the stem cell compartment and restore epithelial tissue homeostasis [17, 62, 86, 128, 205, 206]. However, the inadequate amplification of WNT signaling is associated with the incidence and/or progression of colorectal cancer. A study showed that colorectal cancer is divided into WNT ligand-independent and ligand-dependent subgroups [207]. The former has mutations in the *APC* gene and *CTNNB1* gene encoding *β*-catenin in epithelial cells, which causes constitutive activation of the canonical WNT signaling pathway [208]. Meanwhile, the latter shows the upregulation of Rspo3 expression in fibroblast but lacks epithelial mutations in the *APC* and *CTNNB1* genes.

Regarding findings on mesenchymal stromal cells in the context of cancer, it has been revealed that the interactions of several types of cancer-associated fibroblasts (CAFs) with other cell types are associated with metastasis and cancer progression by facilitating angiogenesis and promoting the accumulation of regulatory T cells and anti-inflammatory tumor-associated macrophages [209]. In patients with colorectal cancer, the augmentation of CAFs secreting the BMP signaling antagonist Gremlin 1 is associated with poor survival, whereas CAF secreting the BMP signaling activator ISLR inhibit colorectal cancer progression [210]. Gremlin 1 expression is upregulated in intestinal epithelial cells of patients with hereditary mixed polyposis syndrome characterized by the development of multiple types of colorectal tumors, which provides Lgr5^−^ cells excluded from the stem cell niche with stem-like properties by disrupting the homeostatic Gremlin 1 gradient at the crypt base-villus axis [211]. Elsewhere, it has been reported that CAFs within *APC*-mutant colorectal cancer secrete a large amount of WNT2 [29, 212, 213], which provokes the expression of extracellular matrix molecules and proangiogenic factors through autocrine WNT signaling via the receptor FZD8 in CAFs and fibroblasts. This in turn leads to the progression of tumor growth and metastasis [213, 214]. Low WNT signaling activity is associated with the induction of CXCL12-producing inflammatory CAFs (iCAFs) that induce expression of the genes involved in epithelial-mesenchymal transition in tumor cells, leading to cancer dissemination [209, 215]. Meanwhile, it has been shown that CXCL12^+^ iCAFs are increased during colorectal cancer progression, and the pathway related to drug metabolism is activated in these cells [216], suggesting that CXCL12^+^ iCAFs are associated with chemotherapy resistance in patients with colorectal cancer. TGF-β-induced suppression of PKCζ activity induces CAFs expressing WNT antagonists SFRP1 and SFRP2 in a SOX2-dependent fashion, which is associated with poor prognosis in colorectal cancer by establishing an immunosuppressive tumor environment [217]. These findings indicate that the WNT signaling-induced generation and expansion of several types of CAFs as well as CAF-mediated modulation of WNT signaling exacerbate the progression of intestinal cancer.

## Conclusion

Mesenchymal stromal cells have been primarily conceptualized as structural cells that maintain the architecture of organs and tissues and the positioning of the cells within them [12, 157, 209, 218, 219]. However, accumulating evidence indicates that these cells also function as key regulators of immune responses and epithelial integrity in the intestine [55, 94, 101, 102, 220, 221]. As summarized in this review, intestinal mesenchymal stromal cells are a heterogeneous population with distinct spatial localizations and functions in different developmental stages, homeostatic conditions, and intestinal diseases. In patients with intestinal diseases, besides alterations in the character of tissue-resident mesenchymal stromal cells, the microbiota community and metabolomic profiles in biological samples are also changed [222, 223]. Microbiota-derived bioactive metabolites exert beneficial and deleterious effects on the host immune system, epithelial barrier, and microbial ecosystems [224–228]. However, the impacts of microbiota-supplied metabolites on mesenchymal stromal cell physiology remain poorly understood. Moreover, the cellular origin of tissue-resident and disease-associated mesenchymal stromal cells and the mechanism regulating their differentiation, localization, and activation remain incompletely defined. There is thus a need for a more detailed and complete fate map of intestinal mesenchymal stromal cell populations and the local signaling molecules to manipulate their differentiation and function both in a steady state and under inflammatory conditions. This will aid the identification of novel targets for the development of medications for IBD and colorectal cancer, as well as other intractable diseases. There is also a need to define how interactions between factors associated with the intestinal environment and IBD susceptibility genes in mesenchymal cells are involved in the maintenance of intestinal homeostasis and/or the pathogenesis of IBD. This might help to unravel some of the complex etiology of IBD.

## Data Availability

Not applicable.

## Abbreviations

APC: APC regulator of WNT signaling pathway
ANGPTL4: Angiopoietin-like 4
BMP: Bone morphogenetic proteins
CAF: Cancer-associated fibroblast
CCL: C–C motif chemokine ligand
Col: Collagen
CXCL: C-X-C motif chemokine ligand
CXCR: C-X-C motif chemokine receptor
DKK: Dickkopf
Dll1: Delta-like 1
EGF: Epidermal growth factor
Fgfr2: Fibroblast growth factor receptor 2
Ereg: Epiregulin
Grem: Gremlin
hDTR: Human diphtheria toxin receptor
Hh: Hedgehog
HHIP: Hedgehog-interacting protein
IBD: Inflammatory bowel disease
IGF: Insulin-like growth factor
IFN-γ: Interferon gamma
IL: Interleukin
Lgr5: Leucine-rich repeat-containing G-protein coupled receptor 5
LRC: Long-term label-retaining cell
Lrp5: LDL receptor-related protein 5
LTβR: Lymphotoxin B receptor
M cell: Microfold cell
MMP: Matrix metalloproteinase
MAPK: Mitogen-activated protein kinase
MAP3K8: Mitogen-activated protein kinase kinase kinase 8
NEC: Neonatal necrotizing enterocolitis
Ng2: Neuron-glial antigen 2
NPNT: Nephronectin
PCP: Planar cell polarity
PGE_2_: Prostaglandin E_2_
Porcn: Porcupine O-acyltransferase
RANKL: Receptor activator of nuclear factor-kappa B ligand
Rspo: R-spondin
scRNA-seq: Single-cell RNA sequencing
Sema3: Semaphorin 3
SGLT1: Sodium-dependent glucose transporter 1
Shh: Sonic hedgehog
TA cell: Transit-amplifying cell
TCF/LEF: DNA-binding T-cell factor/lymphoid enhancer factor
TLR4: Toll-like receptor 4
